# Outcomes of esophagectomy for patients with esophageal squamous cell carcinoma accompanied by recurrent laryngeal nerve palsy at diagnosis

**DOI:** 10.1007/s10388-021-00890-6

**Published:** 2021-10-27

**Authors:** Asako Ozaki, Shinji Mine, Kouhei Yoshino, Daisuke Fujiwara, Motomi Nasu, Tadasuke Hashiguchi, Takashi Hashimoto, Yoshiaki Kajiyama, Masahiko Tsurumaru, Atsushi Arakawa

**Affiliations:** 1 Department of Esophageal and Gastroenterological Surgery, Juntendo University Hospital, Juntendo University, 3-1-3, Hongo, Bunkyo-ku, Tokyo, 113-8431 Japan; 2 Department of Human Pathology, Juntendo University School of Medicine, Tokyo, Japan

**Keywords:** Esophageal cancer, Esophagectomy, Recurrent laryngeal nerve, Hoarseness

## Abstract

**Background:**

Hoarseness is one of the classical symptoms in patients with locally advanced thoracic esophageal squamous cell carcinoma (ESCC), and it results from recurrent laryngeal nerve palsy, which is caused by nodal metastasis along the recurrent laryngeal nerve or by main tumors. We reviewed the short-term and long-term results of esophagectomy for patients with locally advanced ESCC and hoarseness at diagnosis.

**Patients:**

Patients who initially presented with hoarseness from recurrent laryngeal nerve palsy between 2009 and 2018 and underwent esophagectomy for thoracic ESCC were eligible for this study. Pharyngolaryngectomy or cervical ESCC were exclusionary.

**Results:**

A total of 15 patients were eligible, and 14 underwent resection of the recurrent laryngeal nerves. The remaining patient had nerve-sparing surgery. Nine patients (60%) had post-operative complications ≥ Clavien–Dindo class II and, pulmonary complications were most common. Two patients (13%) died in the hospital. The 5-year overall survival rate for all patients was 16%. Age (≤ 65 years), cT1/T2 tumor, and remarkably good response to neoadjuvant treatment were likely related to longer survival; however, these relationships were not statistically significant.

**Conclusions:**

Esophagectomy for ESCC patients who are diagnosed with recurrent laryngeal nerve paralysis at initial presentation could be a treatment option if the patient is relatively young, has a cT1/T2 tumor, or shows a remarkably good response to neoadjuvant treatment. However, clinicians should be aware of the possibility of postoperative pulmonary complications, which were frequently observed with the procedure.

## Introduction

Esophageal cancer was the seventh most common cancer worldwide and the sixth most common cause of death from cancer in 2018, with an estimated 572,034 new cases (3.2% of the total cancer cases) and 508,585 deaths (5.3% of the total cancer deaths) according to the WHO GLOBOCAN database [1]. Esophageal cancer is divided into two major types: adenocarcinoma and squamous cell carcinoma (SCC). In East-Asia, including Japan, SCC is dominant for esophageal cancer.

Although multi-disciplinary treatments for esophageal cancer have become common in the past two decades, esophagectomy with adequate lymphadenectomy remains the mainstay of curative treatment for locally advanced esophageal cancer [2]. In esophageal SCC (ESCC), lymph node metastasis along recurrent laryngeal nerves is very frequent, and lymphadenectomy along the recurrent laryngeal nerves is one of the most critical procedures in esophagectomy [3].

Hoarseness, which is caused by paralysis of either side of the vocal cords, is one of the classical symptoms in patients with locally advanced esophageal cancer. Usually, this paralysis originates from metastatic nodes along the recurrent laryngeal nerves, or sometimes from the main tumors. This symptom is considered to be a poor prognostic indicator, because it could suggest an obvious extranodal invasiveness or an extremely aggressive or invasive cancer [4, 5]. Generally speaking, definitive chemoradiotherapy or systemic chemotherapy has been chosen in these patients because of poor survival.

On the other hand, we have performed aggressive surgical resection for ESCC even if recurrent laryngeal nerve paralysis was found, but a curative resection would be possible. There are few reports of esophagectomy for patients with recurrent laryngeal nerve palsy before treatments [6, 7]. In this study, we retrospectively analyzed the short-term and long-term results of esophagectomy for ESCC with recurrent laryngeal nerve paralysis at diagnosis.

## Patients and methods

From January 2009 to December 2018, 1126 patients underwent esophagectomy for esophageal cancer at the Department of Esophageal and Gastroenterological Surgery, Juntendo University Hospital, Tokyo, Japan. The inclusion criteria for this study were hoarseness at the initial visit, vocal cord paralysis confirmed by an otolaryngologist or endoscopist, and esophagectomy via a right thoracotomy performed for curative intent. Patients who underwent total pharyngolaryngectomy or had cervical esophageal cancer were excluded.

### Surgical procedure

The details of the procedure have been described previously [2]. Briefly, esophagectomy with three-field lymphadenectomy via a right thoracotomy and laparotomy was performed in almost all patients. A recurrent laryngeal nerve causing hoarseness was resected with a responsible node in most patients. In all patients, lymphadenectomy along the contralateral recurrent laryngeal nerve was performed very carefully. A gastric conduit was pulled up in the retrosternal route, and hand–sewn esophagogastric anastomosis was performed in the cervical area. Anastomoses between the resected nerve and the vagal nerve were performed in some patients based on the surgeon’s decisions. When reconstruction of the resected nerve was performed, we anastomosed the end of the recurrent laryngeal nerve to the ipsilateral vagal nerve in an end-to-end manner.

### Clinicopathological parameters

Clinicopathological parameters were retrospectively retrieved from the hospital database, including the tumor stage according to the 8th edition of the TNM classification [8]. Postoperative complications were classified using the Clavien–Dindo classification [9].

### Statistical analyses

All statistical analyses were performed using EZR (Saitama Medical Center, Jichi Medical University, Saitama, Japan), which is a graphical user interface for R software (The R Foundation for Statistical Computing, Vienna, Austria) that incorporates frequently used biostatistical functions [10]. The associations between clinicopathological factors and each group were analyzed using Fisher’s exact test for categorical data or the Mann–Whitney *U* test for continuous variables. Relapse-free and overall survival (OS) rates were estimated using the Kaplan–Meier method and compared using the log-rank test. Differences were considered statistically significant at two-tailed *p* values of < 0.05.

## Results

There were 15/1126 patients with recurrent laryngeal paralysis eligible for this study (1.3%). The characteristics of these 15 patients are shown in Table 1. The primary tumors were located in the upper (6/15, 40.0%), middle (7/15, 46.7%), and lower (2/15, 13.3%) esophagus. Thirteen patients had paralysis from nodal metastases and 2 had paralysis from main tumors. Right paralyses were found in 5 patients, and left paralyses in 10 patients. Five patients received neoadjuvant chemotherapy, 9 patients received neoadjuvant chemoradiotherapy, and 1 patient received no neoadjuvant treatment.

**Table 1:** Patient characteristics

Variables	*N* = 15
Age (median, range)	66 (55–78)
Sex	
Male/female	14/1
Tumor location	
Upper/middle/lower	6/7/2
Neo-adjuvant treatment	
None/chemotherapy/chemoradiotherapy	1/5/9
cT	
T1/T2/T3/T4	2/1/6/6
cN	
N0/N1/N2/N3	0/3/7/5
cM	
M0/M1	9/6
Esophagectomy	
3-fields/2-fields	15/0
Resection of recurrent laryngeal nerve	
With/without	14/1
Operation time (min; median, range)	596 (370–375)
Blood loss (ml; median, range)	635 (190–1220)
Length of ICU stay (days; median, range)	7 (4–77)
Length of hospital stay (days; median, range)	24 (16–328)
pT	
T0/T1/T2/T3/T4	2/3/1/8/1
pN	
N0/N1/N2/N3	2/4/7/2
pM	
M0/M1	10/5
Residual tumor	
R0/R1/R2	13/0/2

### Surgical and pathological findings

Surgical and pathological findings are shown in Table 1. A paralytic recurrent laryngeal nerve was resected in 14 patients, while the nerve was preserved in 1 patient. This patient had histologically positive nodes, and the laryngeal nerve was preserved by sharp dissection along the nerve. Pathological examinations showed that R0 resection was achieved in 13 patients and 2 patients had gross tumor remains (R2). In these pathological diagnoses, R0 resection was performed only for the main tumors, and the pathological margin around the metastatic nodes was not evaluated.

### Short-term outcomes of esophagectomy

Postoperative complications are shown in Table 2. Nine of the 15 patients had postoperative complications of ≥ Clavien–Dindo class II. Pneumonia was the most common and was found in 6 patients. Re-intubations were required in 4 patients and tracheostomy was performed in 2 patients. Two patients had hospital deaths from pneumonia, and their hospitalizations were 116 and 131 days. The median length of postoperative ICU stay was 7 days, and the median length of postoperative hospital stay was 24 days.

**Table 2:** Postoperative complications of esophagectomy

Complications	N = 15
No complication	1
Clavien–Dindo class	
I/II/III/IV/V	5/0/5/2/2
Pneumonia	6
Reintubation	4
Tracheostomy	2
Anastomotic leakage	2
Chylothorax	2
Mortality within 30 days	0

### Paralysis of the vocal cords

All patients showed paralysis of the affected vocal cords, of whom two patients showed bilateral paralysis of the vocal cords. Anastomosis of the recurrent nerve and ipsilateral vagal nerve trunk was performed in 8 of the 14 patients. Of these 8 patients, 3 showed complete paralysis of the affected vocal cord with adequate compensation of the contralateral vocal cord. The other 5 patients had complete paralysis of the affected vocal cord without compensations [7].

### Pathological findings of responsible nodes and the resected nerves

Six patients showed the findings of extranodal infiltration. Of the 14 patients who underwent combined recurrent laryngeal nerve resection, 4 patients showed pathological invasion of the resected nerve. The other 10 showed no pathological findings of nerve invasion. All the 3 patients with pathological findings of invasion of the nerve had extranodal infiltration.

### Long-term outcomes of esophagectomy

**Fig. 1:**
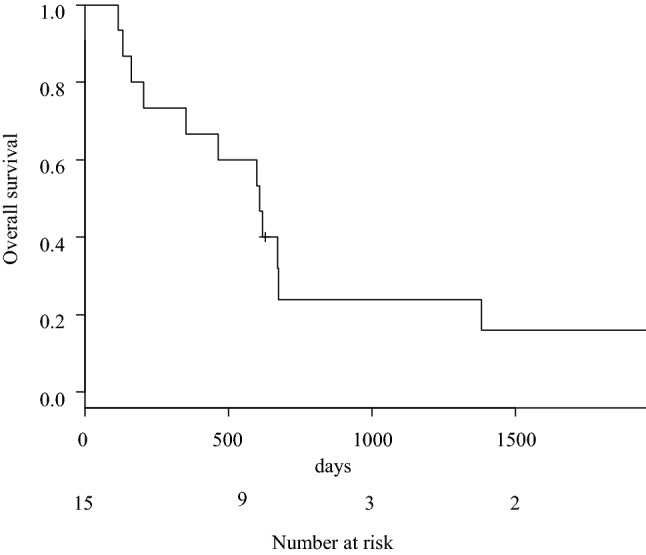
Overall survival among patients who underwent esophagectomy for esophageal squamous cell carcinoma with recurrent laryngeal nerve paralysis at the initial visit

The median OS of the 15 patients was 610 days, and the 5-year OS rate was 16.0% (Fig. 1). The median relapse-free survival was 383 days, and the 5-year relapse-free survival was 17.8%. The survival of the 2 patients with R2 resection was of a short duration, at 131 and 205 days, respectively.

Of the 15 patients, 10 had recurrent disease. Lymph node recurrences were found in 6 patients (neck in 4, mediastinum in 3, and abdomen in 1), pleural dissemination in 1 patient, lung metastasis in 4 patients, and bone metastasis in 1 patient (including duplications).

### Relationships between survival and clinicopathological factors

**Fig. 2:**
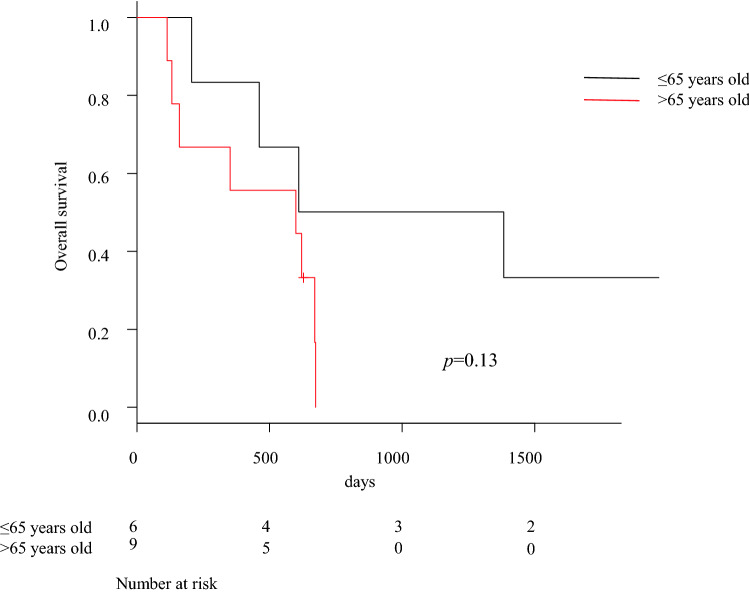
Analysis of overall survival outcomes according to patients’ age

**Fig. 3:**
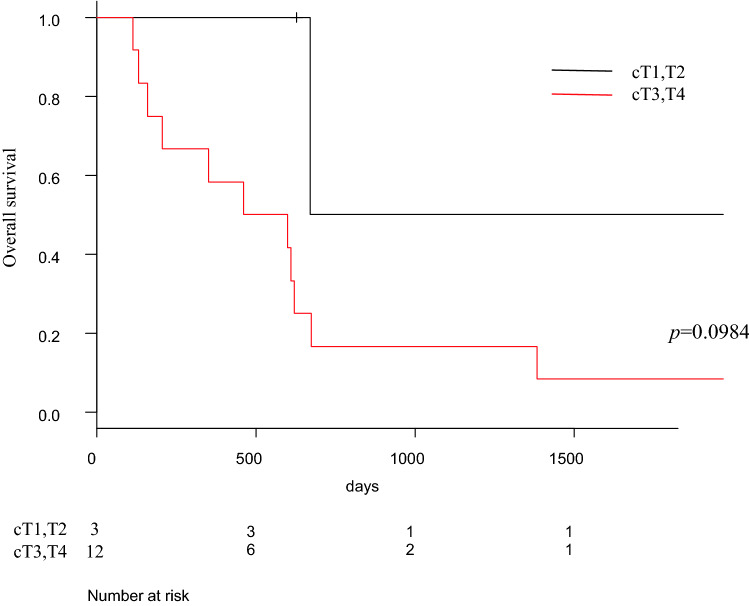
Analysis of overall survival outcomes according to clinical T status

**Fig. 4:**
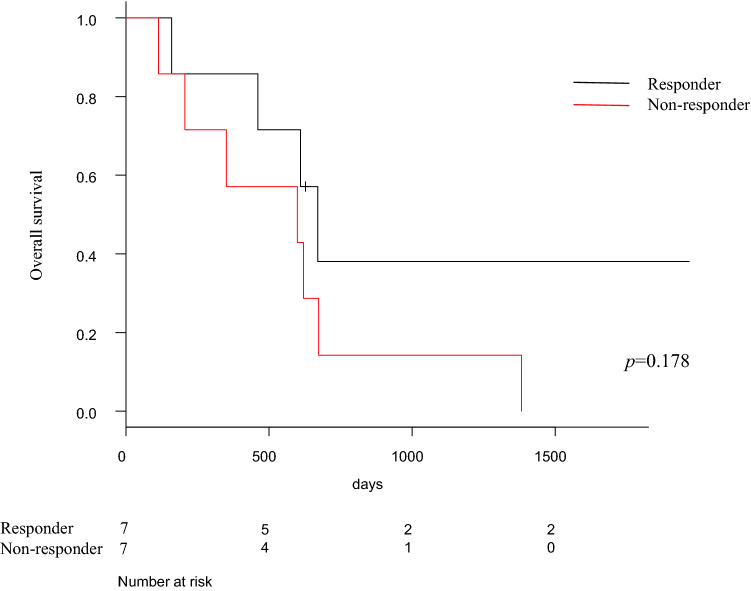
Analysis of overall survival outcomes according to effect of neoadjuvant treatment

The OS of these patients was correlated with several clinical factors. Younger patients (≤ 65 years) and patients with cT1 or T2 tumors were likely to have longer survival, but these associations were not statistically significant (*p* = 0.130, 0.0984, respectively) (Figs. 2, and 3). Of the 14 patients who received neoadjuvant treatments, patients whose responses were evaluated as remarkably good on endoscopy, who were classified into the “responder” group, were likely to have longer survival, but this was also not statistically significant (*p* = 0.178) (Fig. 4).

Next, survival was correlated with pathological factors. Residual tumor grades and pathological T status were significantly associated with survival (*p* = 0.0118 and 0.017, respectively). The pathological number of nodal metastases was likely to be prognostic but was not statistically significant (*p* = 0.12). The presence or absence of direct invasion of the nerves in the pathological findings and the presence or absence of extranodal infiltration were not related to OS (*p* = 0.78. and 0.88, respectively).

Finally, survival was correlated with postoperative complications. None of the complications (Clavien–Dindo class ≥ II) were related to survival (*p* = 0.541). All pulmonary complications (Clavien–Dindo ≥ II) were likely to be associated with shorter survival; however, the association was not statistically significant (*p* = 0.113).

## Discussion

This study retrospectively analyzed the short- and long-term outcomes of esophagectomy for ESCC patients who were diagnosed with recurrent laryngeal nerve paralysis at initial presentation. Because there are very few reports about surgical outcomes, we believe that this study will be significant in determining a strategy for such patients.

Tachimori et al. reported that of 478 consecutive patients with thoracic esophageal carcinoma, 24 patients (5%) had vocal cord paralysis [6]. Of these 24 patients, only two patients underwent radical surgery by esophagectomy with resection of the metastatic lymph node infiltrating the recurrent laryngeal nerve. None of these 24 patients with hoarseness survived without disease for more than 1 year.

Natsugoe et al. reported on 5 patients with preoperative recurrent nerve paralysis undergoing esophagectomy with resection and reconstruction of recurrent laryngeal nerves [7]. They showed that 4 of 5 patients survived more than 1 year with good quality of life, but long-term survival was not shown for this very small number of patients.

In our cohort, the incidence of postoperative complications of Clavien–Dindo class ≥ II was high (9/15, 60%) and two patients (13.3%) had hospital mortality. Of all complications, the incidence of pulmonary complications was the highest, as expected. In addition, we showed that postoperative pulmonary complications were likely related to poor survival. Therefore, efforts should be made to prevent pulmonary complications, such as inducing minimally invasive esophagectomy and peri-operative intensive rehabilitation.

Long-term survival (5-year OS, 16.0%) was poor. However, a previous report showed that the 3-year OS rate for patients who received definitive chemoradiotherapy for cT4b esophageal squamous cell carcinoma was only 14% [11]. In comparison, esophagectomy could be an alternative option for fit patients.

Clinicopathological factors were assessed for relationship to longer survival. Younger patients, cT1 or T2 patients, and good responders to neoadjuvant treatment were likely to have longer survival, although this was not statistically significant. Therefore, esophagectomy could be a treatment strategy for patients who are relatively young, have cT1 or T2 tumors, or are good responders to neoadjuvant treatment. Pathological examinations showed that residual tumor status and pT status were strong prognostic factors, similar to previous studies. Meanwhile, two patients underwent R2 resection, and further underwent combined resection of the laryngeal nerves, and the survival of these 2 patients was dismal. We should emphasize that resection should be stopped intraoperatively when complete tumor resection is impossible.

In this study, we reconstructed the resected laryngeal nerve in eight of 14 patients. All of these patients showed laryngeal nerve palsy, while 3 showed paralysis of the affected vocal cord with adequate compensation of the contralateral vocal cord. The other 5 patients had complete paralysis of the affected vocal cord without compensation. The purpose of the reconstruction of the laryngeal nerve is to prevent atrophy of the affected vocal cord^7^, and we believe that our procedure achieved remarkably good results.

This study has several limitations. First, this was a retrospective and small-size study. Therefore, the statistical power is inadequate to make a significant conclusion. However, we could not find published reports for a large number of patients with hoarseness. Second, our results should be compared to those of patients who received definitive chemoradiotherapy. Unfortunately, there is little data on definitive chemoradiotherapy for these patients to date and comparison to our results was not possible.

In conclusion, esophagectomy for ESCC patients who were diagnosed with recurrent laryngeal nerve paralyses at diagnosis could be a treatment option if the patient is young, has a cT1 or T2 tumor, or shows a good response to neoadjuvant treatment. However, postoperative complications, especially pulmonary complications, were frequently observed, and surgeons should take measures to decrease the risks for these patients.
